# Differential gene expression analysis and SNP/InDel marker discovery in resistant wild *Asparagus kiusianus* and susceptible *A. officinalis* in response to *Phomopsis asparagi* infection

**DOI:** 10.1016/j.dib.2018.11.034

**Published:** 2018-11-13

**Authors:** Mostafa Abdelrahman, Mai Mitoma, Takao Ikeuchi, Mitsutaka Mori, Kyoko Murakami, Yukio Ozaki, Masaru Matsumoto, Atsuko Uragami, Akira Kanno

**Affiliations:** a Arid Land Research Center, Tottori University, Tottori 680-0001, Japan; b Botany Department, Faculty of Sciences, Aswan University, Aswan 81528, Egypt; c Kagawa Prefectural Agricultural Experiment Station, 1534-1 Ayagawa, Ayauta, Kagawa 761-2306, Japan; d Faculty of Agriculture, Kyushu University, Fukuoka 811-2307, Japan; e Institute of Tropical Agriculture, Kyushu University, Fukuoka 812-8581, Japan; f Institute of Vegetable and Floriculture Science, NARO, Tsukuba, Ibaraki 305-8519, Japan

**Keywords:** RNA-Seq, Transcriptome, Asparagus, Single nucleotide polymorphism, Insertion/deletion

## Abstract

This data article reports *de novo* transcriptome analysis of resistant wild *Asparagus kiusianus* and susceptible *A. officinalis* plants 24 and 48 h post-inoculation (24 and 48 hpi) with *Phomopsis asparagi*. Differential gene expression (DGE) analysis demonstrated that several genes involved in secondary metabolites and plant-pathogen interactions are up-regulated in resistant wild *A. kiusianus* relative to susceptible *A. officinalis*. The assembled contig sequences generated in this study were used to search single nucleotide polymorphism (SNP) and insertion/deletion (InDel) distribution in *A. kiusianus* and *A. officinalis* plants. SNP and InDel data developed from this transcriptome analysis will be used to generate a high-density linkage map to facilitate further development of molecular marker-assisted selection in *A. officinalis*.

**Specifications table**

**Table:** 

Subject area	*Biology*
More specific subject area	*Plant molecular biology*
Type of data	*Excel file and figure*
How data were acquired	*Next-generation sequencing using Illumine HiSeq. 2500 platform*
Data format	*Raw, analyzed*
Experimental factors	*Resistant A. kiusianus and susceptible A. officinalis plants were inoculated with P. asparagi and samples were collected 24 and 48 h post-inoculation.*
Experimental features	*Transcriptome analysis was performed using cDNA libraries of A. officinalis and A. kiusianus 24 and 48 hpi with P. asparagi. The assembled contigs were further used for DEG analysis and SNP and InDel discovery.*
Data source location	*Sendai, Japan*
Data accessibility	*SNP and InDel data are included in this article*

**Value of the data**

• This is the first report about transcriptome dynamics and SNP/InDel variants associated with *Phomopsis* disease resistance in resistant wild *Asparagus kiusianus* and susceptible *A. officinalis* plants.
• DEG analysis provides a valuable information about defense responsive genes in *Asparagus* species against *Phomopsis* disease.
• SNP and InDel variants provided in this study will be useful for researchers involved in the future development of high-density linkage maps associated with *Phomopsis* disease resistance.
• SNP and Indel data can be further used for phylogenetic analysis of different *Asparagus* populations.

## 1 Data

**Fig. 1:**
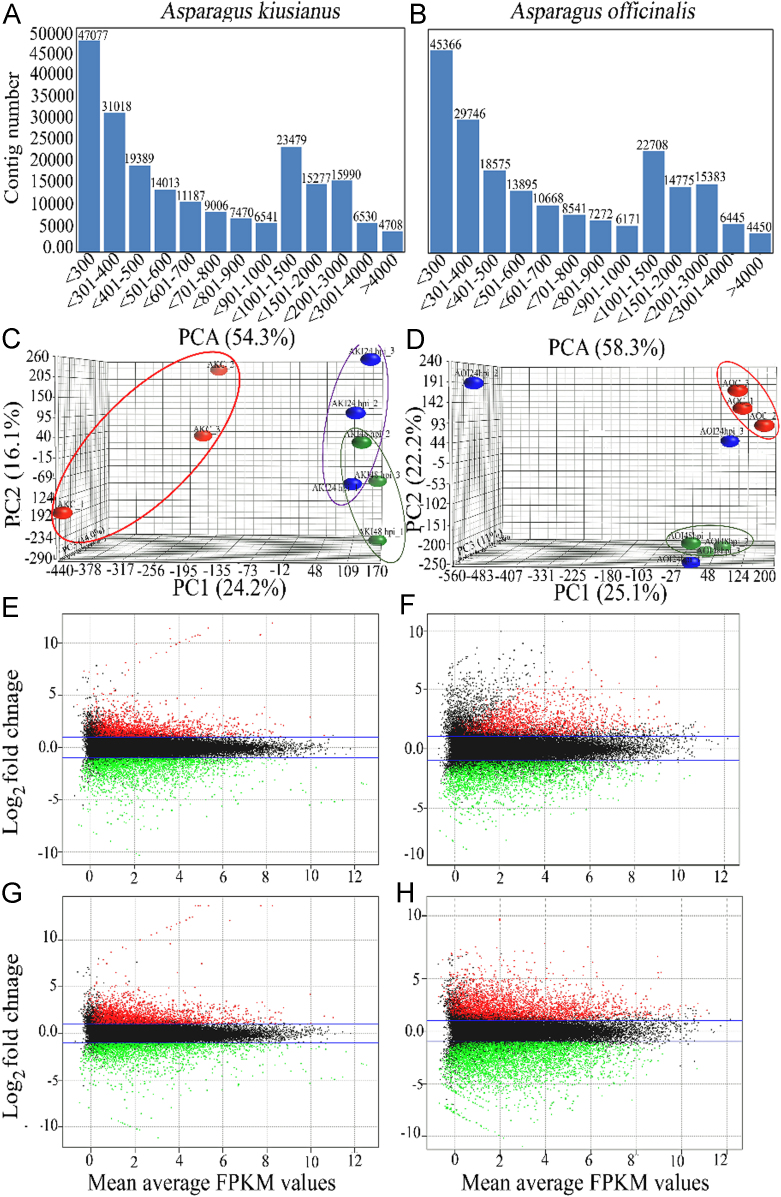
Principal component analysis (PCA) and MA scatter plot of *Asparagus kiusianus* and *A. officinalis* 24 and 48 h post-inoculation (AKI_24hpi, AKI_48hpi, AOI_24hpi, and AOI_48hpi, respectively) with *Phomopsis asparagi* in comparison with non-inoculated control plants (AKC and AOC, respectively). (A and B) Length distribution of assembled transcriptome fragments in *A. kiusianus* and *A. officinalis*. (C and D) PCA analysis of AKI_24hpi, AKI_48hpi, AOI_24hpi, and AOI_48hpi relative to non-inoculated control plants (AKC and AOC, respectively). (E and F) MA scatter plots of differential gene expression in AKI_24hpi and AKI_48hpi in comparison with AKC control plants. (G and H) MA scatter plots of differential gene expression in AOI_24hpi and AOI_48hpi in comparison with AOC control plants. Log2 fold change on the *y*-axis and average count of RPKM (Reads Per Kilobase of exon per Million mapped reads) values on the *x*-axis. Significantly up-regulated genes (red, fold change > 2 and FDR < 0.05), down-regulated genes (green, fold change < 0.5 and FDR < 0.05).

In this study, the plant defense response in resistant wild *A. kiusianus* and susceptible *A. officinalis* was investigated 24 and 48 hpi with *P. asparagi* in comparison with non-inoculated control plants. Our recent study [1] showed that *A. kiusianus*, a wild relative of cultivated *A. officinalis*, displayed significantly reduced disease symptoms compared with susceptible *A. officinalis* upon artificial inoculation with *P. asparagi* [1]. In this study, we conducted *de novo* transcriptome analysis of resistant wild *A. kiusianus* and susceptible *A. officinalis* 24 and 48 hpi with *P. asparagi*. In total, 390,811,866 and 432,232,432 read counts with 100 bp read length were generated from 18 cDNA libraries. After removing adaptors and low-quality reads, more than 98% of the raw reads were clean reads. The high-quality reads were *de novo* assembled using Trinity software, and more than 95.68% and 95.74% were successfully mapped for the *A. officinalis* and *A. kiusianus* samples, respectively. In total, 206,164 and 213,950 contigs (average length, 973 bp) were obtained from *A. officinalis* and *A. kiusianus*, respectively. The quality of transcriptome assemblies was assessed, and the length distribution of the contigs in both *A. officinalis* and *A. kiusianus* is shown in Fig. 1A and B. Principal component analysis (PCA) of the transcriptome data (two *Asparagus* species × three biological replicates × three treatments (control, 24 and 48 hpi)) demonstrated a significant segregation in the wild resistant *A. kiusianus* (accumulation of variance 54,3%) and *A. officinalis* (accumulation of variance 58.3%) 24 and 48 hpi with *P. asparagi* relative to untreated control plants (Fig. 1C and D). The DEG analyses revealed that the total number of up-regulated transcripts in resistant wild *A. kiusianus* (7728) was relatively higher than that (7499) in susceptible *A. officinalis* (Fig. 1E and F). However, down-regulated transcripts (10,713) in susceptible *A. officinalis* was relatively higher than that (6789) in resistant wild *A. kiusianus* (Fig. 1G and H), suggesting that gene expression responses to *P. asparagi* infection differed between susceptible *A. officinalis* and resistant wild *A. kiusianus*. Further, we conducted SNP and InDel distribution in the resistant wild *A. kiusianus* and susceptible *A. officinalis* transcriptome using the recently released *A. officinalis* reference genome in NCBI (Tables S1–S4).

## 2 Experimental designs, materials and methods

Male wild *A. kiusianus* (AK0501 strain) and female *A. officinalis* ‘Mary Washington 500W’ were cultivated under greenhouse conditions at Kagawa Prefectural Agricultural Experiment Station, Kagawa, Japan. Total RNA was extracted from 5-year-old *A. officinalis* and wild *A. kiusianus* 24 and 48 hpi with *P. asparagi* and from non-inoculated *Asparagus* plants grown under the same conditions (two *Asparagus* species × three biological replicates × three treatments (control, 24 and 48 hpi)). RNA concentration and quality were assessed using gel electrophoresis and UV/VIS Beckman DU 730 spectrophotometer (Beckman Coulter Inc., San Diego, CA, USA) and Agilent 2100 Bioanalyzer (Agilent Technologies Inc., USA) instruments. Paired-end reads were generated by TaKaRa Bio (TaKaRa Bio, Kusatsu, Japan) using an Illumina HiSeq. 2500 instrument (Illumina Inc., USA). The raw reads were trimmed using Cutadapt v.1.339 and Sickle v.1.200. After trimming, 26.5 Gb of data were used for *de novo* transcriptome assembly using Trinity package v.2.0.6 [2], [3]. After assembly, 213,950 and 206,164 contigs were obtained from *A. officinalis* and *A. kiusianus*, respectively. These contigs were further used for DEG analysis. Sequencing read counts were calculated using RSEM v1.2.15 [4]. Gene expression from different samples was normalized by the TMM method [5]. DEGs were determined using the edgeR program. Genes with false discovery rate (FDR) < 0.05 and fold change > 2 were considered to be differentially expressed. PCA analysis was carried out by R statistics v3.4 (https://www.r-project.org/) using PCA-based unsupervised gene expression of *A. officinalis* and *A. kiusianus.*

SNPs and InDels between *A. officinalis*, wild *A. kiusianus,* and the recently released *A. officinalis* reference genome were precisely pinpointed using a variant calling process. RNA-Seq reads were aligned to the reference genome using TopHat v. The output BAM files were subjected to SNP/InDel calling using PICARD and GATK (http://www.broadinstitute.org/gatk/) using the default parameters. In each condition, SNPs with reading depth > 5 and quality > 20 were identified as putative homozygous SNPs. The read depth at each locus was calculated using BED tools.
